# Correction: Freitas et al. Influence of Climate Change on Chestnut Trees: A Review. *Plants* 2021, *10*, 1463

**DOI:** 10.3390/plants11111518

**Published:** 2022-06-06

**Authors:** Teresa R. Freitas, João A. Santos, Ana P. Silva, Hélder Fraga

**Affiliations:** 1 Research Centre for the Research and Technology of Agro-Environmental and Biological Sciences (CITAB), University of Trás-os-Montes e Alto Douro (UTAD), P.O. Box 1013, 5000-801 Vila Real, Portugal; jsantos@utad.pt (J.A.S.); hfraga@utad.pt (H.F.); 2 Research Centre for Agricultural Sciences and Engineering, Department of Agronomy, University of Trás-os-Montes e Alto Douro (UTAD), P.O. Box 1013, 5000-801 Vila Real, Portugal; asilva@utad.pt

In the original article [1], there were some misinterpretations of the FAO dataset regarding *Castanea* production that impacted most of the data from Bolivia. The available chestnut production information in the FAO dataset included data on not only the genus *Castanea* but also *Bertholletia excelsa* Humb. & Bonpl. To our knowledge, this was due to the translation of the Spanish words “Castaña del Brasil” (literally Chestnut from Brazil) into English, which corresponded to *Bertholletia* *excelsa*. Therefore, these misinterpretations must be taken into consideration in Table 1 and the corresponding text of the article.

Furthermore, in the sentence “In the Mediterranean region, the water is increasing due to climate change” in Section 4.1.1. Water Management, the word “requirements” is missing. The correct sentence is: “In the Mediterranean region, the water requirements are increasing due to climate change”. The absence of the word in the sentence changes its meaning.
